# Round the Hospitals

**Published:** 1919-05-24

**Authors:** 


### ROUND THE HOSPITALS.

The King has been pleased to award the Royal Red Cross to the following ladies in recognition of their valuable nursing services during the war:—Bar to the Royal Red Cross, Miss Margaret F. Steele, R.R.C., Matron Prince of Wales' Hospital for Officers; Miss S. L. Wilshaw, R.R.C., Matron Military Hospital, Bangor. Royal Red Cross, First Class: Miss M. E. Webster, Q.A.I.M.N.S.R. Second Class: Assistant Matron E. Clarke; Assistant Matron E. L. Huggins; Staff Nurse Marion MacDall; Staff Nurse A. E. Midgley. The following awards were made for valuable services under the British Red Cross Society or the Order of St. John: Royal Red Cross, First Class: Miss Mabel Campbell, Matron; Sister C. Dover; Miss M. M. Innes, Commandant and Lady Superintendent; Miss Flora Masson, Matron. Second Class: Miss Blanche Ormerod, Sister-in-Charge.

**ROUND THE HOSPITALS**—(*continued*).

At the Investiture held in the Quadrangle of Buckingham Palace on May 15 the King personally conferred the following decorations on members of the nursing services:—Royal Red Cross, First Class, Sister Kate Austen, St. John Ambulance Brigade; Second Class: Matron M. Barwell, Sister Frances Barwell, and Staff Nurse Lydia Abell, Q.A.I.M.N.S.R.; Sister E. Woodhouse and Staff Nurse Anna McLeod, T.F.N.S.; Sister E. Blake, Sister E. Burgess, Sister E. Pearson, Sister B. Thornton, Sister D. Van Nickerk, Sister I. Waters, and Staff Nurse E. Currie, South African M.N.S. Military Medal for Bravery in the Field: Sister Sarah Johnson, Q.A.I.M.N.S.R. Miss Evelyn Boake, Miss E. Mooney, and Mrs. Rawdon Smith, V.A.D., also received the Second Class Royal Red Cross from the King, and Miss E. Faulder, Miss S. Bonnell and Miss A. Faulkner, First Aid Nursing Yeomanry, the Military Medal. Queen Alexandra subsequently received the above ladies at Marlborough House.

The College of Nursing announces that arrangements have been made to afford members gratuitous legal advice. It frequently happens that nurses are placed, without any fault of their own, in predicaments from which they cannot escape without risk of financial loss unless they act under good and disinterested legal advice. Ignorance and the fear of incurring heavy fees often restrain them from approaching a good solicitor, and an incalculable amount of worry, accompanied not seldom with the surrender of lawful claims, are the result. Members of the College may now state any legal difficulties encountered in the course of their professional work as clearly as they can in a communication addressed to the Secretary of the College, and will receive a legal opinion, free of charge, given by a competent lawyer. Of course, the College of Nursing cannot undertake to pay the nurses' expenses should it be desirable for her to prosecute. She will be told what is best for her to do, and this is the really important matter.

The fourth annual report of the College of Nursing reveals the College as a thoroughly live and vigorous association. Young societies must devote the major part of their energies to the business of increasing their membership and consolidating their organisation. In both these directions the College has done splendidly during the past year. Its membership is now over 13,000, and its local centres number nineteen. It has entered upon permanent premises at 7 Henrietta Street, has afforded substantial aid to demobilised nurses through its Appointments Bureau, and has taken steps to prepare the register of its members for publication. The value of all the spade work which has gone to make up these items in the Report far outweighs that of its "representation on organised bodies" and the resolutions it has forwarded to high quarters, although we would not be thought to depreciate these symptoms of energy.

That it is the most pressing business of the young Society to grow may be seen by the stress rightly laid upon the number of nurses attached to the College in the controversies over the Registration Bill. The moral of all the talking lies for the individual nurse in her duty first to join the College herself and next to draw in her friends. When the numbers rise to 20,000 strong the momentum will beat down all factious opposition. Members should read the lines in the report dealing with the Tribute Fund with particular attention. Every increase of membership widens the general responsibility for members who are sick and in need, and though membership of the College is not an essential preliminary to receiving aid, it is a means whereby nurses come to a knowledge of what the College is doing for nurses. One grateful nurse says, "I believe the Tribute Fund is very little known among nurses generally." The only way to make it better known is for the College to grow. It is highly satisfactory that the treasurer's report is able to show a substantial balance over and above the £32,500 received from the Nation's Fund for Nurses. The accumulated fund now shows a total of £42,428.

The visit of the Queen and Princess Mary to the Shenfield Schools of the Poplar Board of Guardians on May 14 is one of those incidents which let a flood of light into the long, slow labours of the pioneers. What the barrack schools were of some twenty or thirty years ago many of us vividly remember. The vacant faces and listless movements of the pauper children were a blot on the nation it was easier to perceive than to remove. Between that waste of human faculties and the admirable organisation which the Queen inspected with trained thoroughness lies a period of reconstructive work paralleled, perhaps, in other directions, but in none outdone. Seven hundred boys and girls can be received at Shenfield, and in some classes co-education is followed. Consideration of the collective result and its effect on the future of the nation is an excellent antidote to pessimism. The keynote was sounded in a fine rendering of Sir Hubert Parry's setting of Blake's verses, the solo being taken by a little boy of ten, and all the children joining in the chorus, "Till we have built Jerusalem in England's green and pleasant land."

Commemoration Day, May 30, is to be kept in this country with appropriate rites this year. Our readers will remember that this is the American Festival of the dead who have fallen in the service of their country. Some two thousand officers and men of the American army and navy lie buried in this country, and every grave will be honoured with wreaths and flags provided by the American Red Cross Society. We know far too little about the American nursing sisters who gave their lives in this country in the cause of freedom. There should not be wanting some ceremonial observance of their memory on May 30, and the need for some centrally situated church, permanently associated with nurses, such

**ROUND THE HOSPITALS—(*continued*).**

as the Rev. A. Lombardini has been talking about recently, makes itself strongly felt in this connection. Such a church would contain a memorial of all those who fell in the War, as well as of those who from day to day give their lives for others in the ordinary course of duty, and would form a centre for commemoration and for the corporate worship of the nursing profession.

St. Mary's Hospital records an important nursing advance in the annual report just issued. Hitherto nurses have been examined in practical nursing for their certificates by the matron, under whose direction the training is of course conducted. Under the new arrangement the examination will be conducted by an examiner not attached to the hospital. This plan, now adopted in the majority of good training-schools, relieves the matron of an onerous task, which we do not hesitate to affirm ought never to be placed on her shoulders. Among the many good reasons for engaging an outside nursing examiner, not the least is the relief such a plan affords to the matron. We have never yet had it brought to our notice that in any hospital the fee per head paid to an examiner for setting and correcting the papers and conducting the *viva voce* and practical part of the examination ever found its way to the matron when she was required to undertake these duties.

It is greatly to be hoped that the example offered by the Brompton Hospital for Consumption in arranging a fourth-year course for nurses to embrace preparation for public health examinations may be widely followed. The course of free fortnightly lectures at this hospital, open to trained nurses and social workers, is now drawing to a close. The final lecture will be given on May 27, on "Past, Present, and Future," by Miss Gladys Bompas. The course has opened up many fresh lines of inquiry, and has stimulated practical observation and study in more directions than it is possible to estimate. The course has led the way as an illustration of the method to be employed in utilising hospital training as a preparation for public-health work. The certificate granted by the hospital after a year's practical work in the wards and outpatient departments at Brompton, together with the supplementary public health certificates obtainable during the same period, enable trained nurses at once to take up municipal work on the completion of their institution training. It is eminently unfair and unreasonable that after four years' hard work they should have to start training over again.

Lady Algernon Gordon Lennox expresses her gratitude in the Press for the charming gift and address presented to her by the members of "Princess Victoria's Rest Clubs for Nurses" in France, including many sisters from casualty clearing stations and the base, as well as nursing V.A.D. members and other workers behind the lines. The work of organising these clubs was no light task, and their effect in affording relief and saving tired nurses from a breakdown appreciably aided in keeping the great machine well oiled. Lady Blanche speaks with gratitude of the "happy atmosphere created by the lady superintendent" during the four years' work of organising the clubs under the administration of the O.G.M.S.

According to Dr. Whitehouse, Medical Officer of Health at Deptford, there is a great dearth of applicants among trained nurses for posts in connection with health visiting and child welfare. The Deptford Borough Council is advertising for five nurses for public health employment, and offers salaries commencing at £140 a year, rising to £170. Application must be made before June 10 on forms to be obtained at the Town Hall, New Cross Road, S.E. 14. The reason, it may be safely conjectured, why so few trained nurses put in for these advantageous municipal posts is that they lack the necessary training for their duties. It is a grievous stain on nursing organisation that a woman after four years' close application to learning her business in a first-rate training-school is in a worse position for obtaining municipal work than one who has spent a year or eighteen months attending lectures and getting up facts for the examinations which are the passport to this branch of work. Unless some change in the curriculum can speedily be devised which shall enable these examinations to be taken by any who wish to do so during their fourth year, the result will be that municipal authorities will cease to look to the nursing profession to supply their needs. How many demobilised nurses have any acquaintance with the routine work and public conditions of health visiting? Yet, as Dr. Whitehouse says, it is these women who are wanted, and it is deplorable that by a vital defect in training they are placed out of the running.

The special tuberculosis journal for nurses, the inception of which in Austria we lately noticed, contains a paper on the lowest age of entry for nursing, by one Hanna Katz. It appears that the minimum age for probationers was fixed by a ministerial decree at eighteen years. The writer thinks, however, that this is too low, and should be changed to twenty-four, or at least to twenty. Her chief argument is the familiar one that young women of twenty and over are more mature and capable, as well as more likely to have made up their minds properly, and to have realised the responsibilities and hard tasks, as well as the advantages, of their lot as nurses. In this country opinion sets very decidedly in favour of lowering the age of candidates.

Under a recent Army Order members of Q.A.I.M.N.S. who retire on retired pay will be permitted to wear uniform when re-employed in a military hospital, and when attending ceremonies and other functions at which officers appear in uniform when they desire to do so. In resuming the use of uniform on such occasions retired Army

**ROUND THE HOSPITALS—(*continued*).**

nurses are required to wear the letter R on the right-hand corner of the red cape. There is an astonishing gulf between the nurse in mufti and the nurse in uniform at important functions, and the concession is one which will be appreciated by a wide circle.

A LIST of Canadian nurses, to the number of forty-three, who have made the supreme sacrifice and given their lives for their country, is published in the April number of the *Canadian Nurse*, compiled from particulars furnished by Miss Rayside, Matron-in-Chief in Canada. Fourteen of the number were drowned at sea, one was "killed at sea," five died of wounds, and one was killed in action. A list is also given of Canadian recipients of the Royal Red Cross up to June 1918. Forty-three matrons and nursing sisters were granted the first class of the Order and 149 the second class. The number of Canadians in addition who have received the Order since last June is very large.

MISS MARIAN BARWELL, Matron of Yarmouth Hospital, who received the Royal Red Cross at the Investiture on May 15, served right through the War without a break, having been called up on August 5. One of her hospital stories has found its way into the *Evening News*, and is too good to be forgotten:—It was in the earliest days, and they were ordered to evacuate a hospital at very short notice. One of the sisters was of Scottish descent and economical turn of mind. The patients' rice puddings were in the oven, and though the Germans were on the edge of the town she had the puddings taken out, placed on a stretcher, and covered with a cloth. The stretcher-bearers arrived at the station, and a group of high French officers, thinking it was a soldier's body, stood at the salute—while the rice puddings passed.

THE "ragging" incident at a Metropolitan Infirmary is the first case of the kind brought to our notice. The victim was Nurse Emily Russell, and her assailants were six probationers, who admitted having taken her from her bed and forcibly put her into a tepid bath previously prepared for the purpose. She was held under, and cold water was poured on her head. There was no contention that the affair was regarded as a joke by the assailants. They appear to have believed that Nurse Russell commented on them to the patients, and took this mode of signifying their displeasure. They were charged with assault before Mr. Gill at the Tower Bridge Police Court, and proffered an apology, which Nurse Russell declines to accept. The case has been adjourned in the hope that some compromise may be reached, the summons against one probationer, who was apparently only an onlooker, being dismissed.

AMONG the nurses returning war-weary and crowned with honours to Melbourne is Sister Gillingham, who left home on a pleasure trip in July 1914, went to Serbia to nurse in the typhus epidemic, was taken prisoner by the Austrians, and after incredible hardships, during which she was reported dead, arrived safely at last in England. Thence, after a short rest, she departed for service in Egypt, where she served till last year. Truly an unexpected kind of pleasure trip. Sister Helen Tait, A.R.R.C., of the Alfred Hospital, has now returned after four years' active service in Egypt and France. Matron Gertrude Davis, R.R.C., also of the Alfred Hospital, was awarded the Kaisar-i-Hind medal, first class; it is the highest order that can be won by a woman in India, and only one other matron has obtained it. Other Melbourne recipients of the R.R.C. are Matron Alma Bennet and Matron A. E. Dowsley, both for services in India; Matron E. L. Horne and Senior Sister Ethel Butler have obtained the Second Class of the Order. We regret to learn of the death last February of Matron Florence Mackenzie, of the Cue Hospital, W.A., shortly after her return from active service. Prior to her war work she was matron for eight years at the Mount Morgan Hospital, Queensland.

AT the Investiture held in the Quadrangle of Buckingham Palace on May 17 the King personally conferred the decoration of the Royal Red Cross on the following members of the nursing services:—First Class, Sister Jessie Buchanan and Sister Maude Proctor, Australian A.N.S.; Second Class, Staff Nurse Gwenllian Morgan, late T.F.N.S.; Matron Eleanor Marks, B.R.C.S.; Sister Elizabeth Porteous, New Zealand A.N.S.; also Miss O. Bowden, Miss E. Court, and Mrs. Hughes, V.A.D. His Majesty conferred the Military Medal for bravery in the field on four members of the First-Aid Nursing Yeomanry—Mrs. Hailes, Miss G. Peyton-Jones, Miss G. Mosely, and Miss M. Richardson. All the above ladies were received by Queen Alexandra at Marlborough House subsequently.

THE price of shoe-leather in these days gives food for thought to most people with limited incomes, and the purchase of necessary footwear becomes often a difficulty for the light purse. The nurses who work for the Cape Hospital Board must have received the news with much satisfaction that henceforth their uniform allowance would include two pairs of shoes each year. Nurses' conditions of service are largely governed at the Cape under an Act which set up a Provincial Commission for dealing with these matters in respect of all hospital boards. Another new and welcome provision is that for the first time pensions are paid to nurses upon their retirement through age or ill-health. The Cape Hospital Board controls five hospitals, two convalescent homes, two dispensaries, a maternity home, and a district nursing organisation. Thus the whole range of nursing work is covered by one organisation, and to be a general officer or a member of the Board must afford a very liberal education in nursing matters.

